# An open-label proof-of-concept study of intrathecal autologous bone marrow mononuclear cell transplantation in intellectual disability

**DOI:** 10.1186/s13287-017-0748-2

**Published:** 2018-01-31

**Authors:** Alok Sharma, Hemangi Sane, Nandini Gokulchandran, Suhasini Pai, Pooja Kulkarni, Vaishali Ganwir, Maitree Maheshwari, Ridhima Sharma, Meenakshi Raichur, Samson Nivins, Prerna Badhe

**Affiliations:** 1 Department of Medical Services, NeuroGen Brain and Spine Institute, Plot No. 19, Sector 40, Opp Rail Vihar, Next to Seawood Station (w), Navi Mumbai, 400706 India; 2 Department of Research and Development, NeuroGen Brain and Spine Institute, Plot No. 19, Sector 40, Opp Rail Vihar, Next to Seawood Station (w), Navi Mumbai, 400706 India; 3 Department of Neurorehabilitation, NeuroGen Brain and Spine Institute, Plot No. 19, Sector 40, Opp Rail Vihar, Next to Seawood Station (w), Navi Mumbai, 400706 India

**Keywords:** Intellectual disability, Autologous bone marrow mononuclear cells, Stem cells, Cellular therapy, Autologous transplantation, Neurorehabilitation, Positron emission tomography-computed tomography scan

## Abstract

**Background:**

The underlying pathophysiology in intellectual disability (ID) involves abnormalities in dendritic branching and connectivity of the neuronal network. This limits the ability of the brain to process information. Conceptually, cellular therapy through its neurorestorative and neuroregenerative properties can counteract these pathogenetic mechanisms and improve neuronal connectivity. This improved networking should exhibit as clinical efficacy in patients with ID.

**Methods:**

To assess the safety and efficacy of cellular therapy in patients with ID, we conducted an open-label proof-of-concept study from October 2011 to December 2015. Patients were divided into two groups: intervention group (*n* = 29) and rehabilitation group (*n* = 29). The intervention group underwent cellular transplantation consisting of intrathecal administration of autologous bone marrow mononuclear cells and standard neurorehabilitation. The rehabilitation group underwent only standard neurorehabilitation.

The results of the symptomatic outcomes were compared between the two groups. In the intervention group analysis, the outcome measures used were the intelligence quotient (IQ) and the Wee Functional Independence Measure (Wee-FIM). To compare the pre-intervention and post-intervention results, statistical analysis was done using Wilcoxon’s matched-pairs test for Wee-FIM scores and McNemar’s test for symptomatic improvements and IQ. The effect of age and severity of the disorder were assessed for their impact on the outcome of intervention. Positron emission tomography-computed tomography (PET-CT) brain scan was used as a monitoring tool to study effects of the intervention. Adverse events were monitored for the safety of cellular therapy.

**Results:**

On symptomatic analysis, greater improvements were seen in the intervention group as compared to the rehabilitation group. In the intervention group, the symptomatic improvements, IQ and Wee-FIM were statistically significant. A significantly better outcome of the intervention was found in the paediatric age group (<18 years) and patients with milder severity of ID. Repeat PET-CT scan in three patients of the intervention group showed improved metabolism in the frontal, parietal cortex, thalamus, mesial temporal structures and cerebellum. No major adverse events were witnessed.

**Conclusions:**

Cellular transplantation with neurorehabilitation is safe and effective for the treatment of underlying brain deficits in ID.

**Trial registration:**

ClinicalTrials.gov NCT02245724. Registered 12 September 2014.

## Background

In *The Diagnostic and Statistical Manual of Mental Disorders*, fifth edition (DSM V), intellectual disability (ID) has been defined as “a disorder with onset during the developmental period that includes both intellectual and adaptive functioning deficits in conceptual, social, and practical domains” [1]. The prevalence of ID is approximately 1–3% with a corresponding intelligence quotient (IQ) < 70 [2]. The epidemiology of ID suggests that in adults the female-to-male prevalence ratio ranges between 0.7:1 and 0.9:1, while it varies between 0.4:1 and 1:1 in children and adolescents [3]. The pathophysiology leading to ID is poorly understood in almost one-third of diagnosed ID [4]. The onset of disabilities suggests an anomaly in the natural course of brain development, particularly the regions that are associated with higher cognitive functions. The clinical presentations in ID are diverse depending upon the severity of the disability and the underlying cause for the disability [5]. The mechanism of injury involves abnormalities in dendritic branching and connectivity of the neuronal network which limits its ability to process information, especially in early childhood, during which learning and acquisition of intellectual abilities and emotional behaviour occurs [6]. The conventional management strategies involve medications, behavioural therapy, psychological intervention and occupational therapy which aim at stabilising the symptomatic representations in ID [7]. These strategies, however, do not address the underlying neuronal damage.

Recently, cellular therapy has shown safety and efficacy in several neurological disorders [8–11]. Evidence suggests that the stem cells carry out a reparative process through their neuroprotective and neurorestorative properties. Conceptually, the mechanism of action of stem cells should counteract the underlying neuronal network abnormalities in ID and yield beneficial clinical effects in patients [4].

The aim of this study is to assess the safety, efficacy and clinical effects of autologous bone marrow mononuclear cell (BMMNC) intrathecal transplantation in patients with ID.

## Methods

### Ethics statement

Patients were selected based on the World Medical Association Helsinki Declaration for Ethical Principles for medical research involving human subjects [12]. The Institutional Committee for Stem Cell Research and Therapy (IC-SCRT) reviewed and approved the protocol of the study. The intervention was explained to the parents in detail along with possible adverse events. Written informed consent was obtained from the parents of the patients. The consent was also video recorded.

### Study design

The study was designed and conducted as an open-label proof-of-concept study in a single hospital centre, Mumbai, India, starting from October 2011 to December 2015. A total of 58 patients with ID were included in the study. They were divided into the intervention group (*n* = 29) and the rehabilitation group (*n* = 29). The intervention group underwent cellular transplantation and standard neurorehabilitation. The cellular transplantation consisted of intrathecal administration of autologous bone marrow mononuclear cells. Neurorehabilitation included special education, psychological, occupational and speech therapy.

#### Intervention group

- Patient selection criteria
  The inclusion criteria were diagnosed cases of intellectual disability based on the DSM V criteria. The exclusion criteria were presence of acute infections, human immunodeficiency virus (HIV)/hepatitis B virus (HBV)/hepatitis C virus (HCV), malignancies, bleeding tendencies, pneumonia, renal failure, severe liver dysfunction, severe anaemia (haemoglobin < 9), any bone marrow disorder, space-occupying lesion in the brain, any other acute medical conditions such as respiratory infection and pregnant or lactating females.
- Intervention
  i. Pre-intervention assessment: before the intervention, all of the patients underwent a detailed neuroevaluation along with serological, biochemical and haematological tests. Positron emission tomography-computed tomography (PET-CT) scan, magnetic resonance imaging (MRI) and electroencephalogram (EEG) were performed prior to the cellular therapy. Granulocyte colony stimulating factor (G-CSF) injections were administered 72 and 24 hours prior to the procedure.
  ii. Procurement and isolation of autologous BMMNCs: bone marrow aspiration was performed under sedation with local anaesthesia. Bone marrow, 80–100 ml depending on the age and body weight of the patient, was aspirated from the anterior superior iliac crest using the bone marrow aspiration needle and was collected in heparinised tubes. The bone marrow samples were analysed qualitatively and quantitatively using Leishman’s stains to rule out pre-existing malignancy if any and to ensure that the sample is representative of normal bone marrow. The BMMNCs were separated from the aspirate using the density gradient method. Bone marrow was diluted in the ratio of 1:1 with normal saline. The diluted bone marrow was subjected to density gradient separation using Ficoll-Paque media by centrifuging it at 440 × *g* rpm for 35 minutes in a swinging bucket rotor without a brake at 20 °C. MNCs are obtained as a buffy coat. The MNCs were washed three times with normal saline by centrifuging at 300 × *g* for 15 minutes in a swinging bucket rotor without a brake at 20 °C and finally resuspended in 1 ml of normal saline. Manually, the cell viability was calculated using Trypan Blue dye which was confirmed by TALI machine using propidium iodide. The average total number of cells injected was 1.022 × 10^8^ cells with an average cell viability of 96%. CD34^+^ counting was done by fluorescence activated cell sorting (FACS) using CD34 PE antibody (BD Biosciences) and the average count was found to be 292.97 ± 33.2 cells/μl.
  iii. Transplantation of bone marrow mononuclear cells: the separated autologous BMMNCs were immediately injected intrathecally using a 25-gauge spinal needle between the fourth and fifth lumbar vertebrae. Simultaneously, 20 mg/kg body weight of methyl prednisolone in 500 ml Ringer lactate was given intravenously to enhance survival of the injected cells [13]. Patients were then monitored for any procedure-related adverse events.
- Neurorehabilitation: after the transplantation, all patients in the intervention group were provided with personalised standard neurorehabilitation for 4 days. A home rehabilitation programme was planned for each patient depending on the assessment done before the treatment. The programme included psychological intervention, occupational therapy, speech therapy and special education.

#### Rehabilitation group

- Patient selection
  The rehabilitation group included patients with ID who were registered in the outpatient department (OPD). They were undergoing occupational therapy, special education, speech therapy and cognitive therapy. The patients were followed up after 6 months of their OPD sessions and were assessed for symptomatic changes in their condition.
- Rehabilitation regime
  The patients in this group underwent the standard rehabilitation regime that included psychological intervention, occupational therapy, speech therapy and special education.

#### Methodology of analysis

- Intergroup analysis
  The percentage improvements in the symptoms and the degree of improvements were compared between the intervention and rehabilitation groups. A grading system was devised to evaluate and compare the functional outcome in patients of each group as follows: mild improvement, improvement seen in less than 25% of symptoms; moderate improvements, improvements seen in 25–50% of symptoms; and significant improvements, improvements seen in more than 50% symptoms. This was done to distinguish between the effect of the cellular therapy along with multidisciplinary rehabilitation (intervention group) and that of rehabilitation alone (rehabilitation group).
- Intragroup analysis
  A detailed analysis was carried out to study the outcome of the intervention.
  i. Objective scales
    Intelligence quotient (IQ) and Functional Independence Measure (FIM/Wee-FIM) were used as outcome measures to determine the changes in cognitive and adaptive skills and functional improvements. A few of the patients with ID could not perform on the IQ tests as their cognitive abilities were too significantly limited to even understand the questions or the tasks assigned. Therefore, the level of severity of the disability was determined based on the patient’s clinical picture and adaptive functioning in daily life. According to the DSM V, the patient’s level of ID was judged to be mild, moderate or severe based on three domains—conceptual, social and practical—taking into consideration the deficits in general mental abilities needed for functioning in everyday life. The ranges for severity levels of ID based on the IQ score/range were as follows: mild ID, IQ range between 55 and 70; moderate ID, IQ range between 40 and 55; and severe ID, IQ range between 25 and 40.
  ii. PET-CT scan of the brain
    Three patients gave consent to perform repeat PET-CT scan of the brain after 6 months of cellular therapy. The pre-cellular therapy and post-cellular therapy scans were compared to assess the metabolic changes in the brain.
  iii. Statistical analysis
    McNemar’s test was used to establish significance of association between the intervention and the symptomatic improvements as well as IQ. The difference between pre-intervention and post-intervention scores of FIM/Wee-FIM was compared using Wilcoxon’s matched-pairs signed-rank test to find its significance.
  iv. Adverse events
    During the stay in the hospital, signs and symptoms of any allergic reaction were monitored at regular intervals. Long-term major and minor adverse events were monitored to establish the safety of stem cell transplantation. A detailed history was also taken to rule out the presence of any seizures.
  v. Factors affecting the outcome of cellular transplantation in the intervention group:
    analysis was performed to study the effect of age and severity of ID on the clinical outcome of the intervention. The patients were divided into age groups of < 18 years (paediatric) and > 18 years (adult). The effect of severity was determined by comparing the degree of improvements between mild, moderate and severe ID.

## Results

### Description of the sample

A total of 58 patients were included in the study.

Twenty-nine patients with ID were included in the intervention group, with 18 (62.07%) males and 11 (37.93%) females. The age of the population ranged from 4 to 42 years with a mean age of 17.79 ± 7.22 years (Table 1). They were diagnosed on average 6.32 ± 8.43 years before the intervention. The baseline IQ scores ranged from 28 to 72.5 with a mean of 50.25, and FIM scores ranged from 18 to 110 with a mean of 72.93. The total population was divided into mild ID (*n* = 11), moderate ID (*n* = 13) and severe ID (*n* = 5) based on the IQ score (DSM V).

**Table 1:** Demographical data of the patients

		Intervention group	Rehabilitation group
Sex	Males	18	22
	Females	11	7
Age	Average age (years)	17.79 ± 7.22	18.37 ± 8.43
	<18 years (paediatric)	16	16
	>18 years (adults)	13	13
Schooling	Stopped	3	3
	Special schooling	11	21
	Normal schooling	2	3
	No schooling	13	2
Developmental milestones	Normal	4	3
	Delayed	25	26

Twenty-nine patients with ID were included in the rehabilitation group, with 22 (75.86%) males and 7 (24.14%) females. The age of the patients ranged from 4 to 45 years with a mean age of 18.37 ± 9.23 years (Table 1).

#### Intergroup analysis

##### Symptomatic improvements in the intervention group

**Fig. 1:**
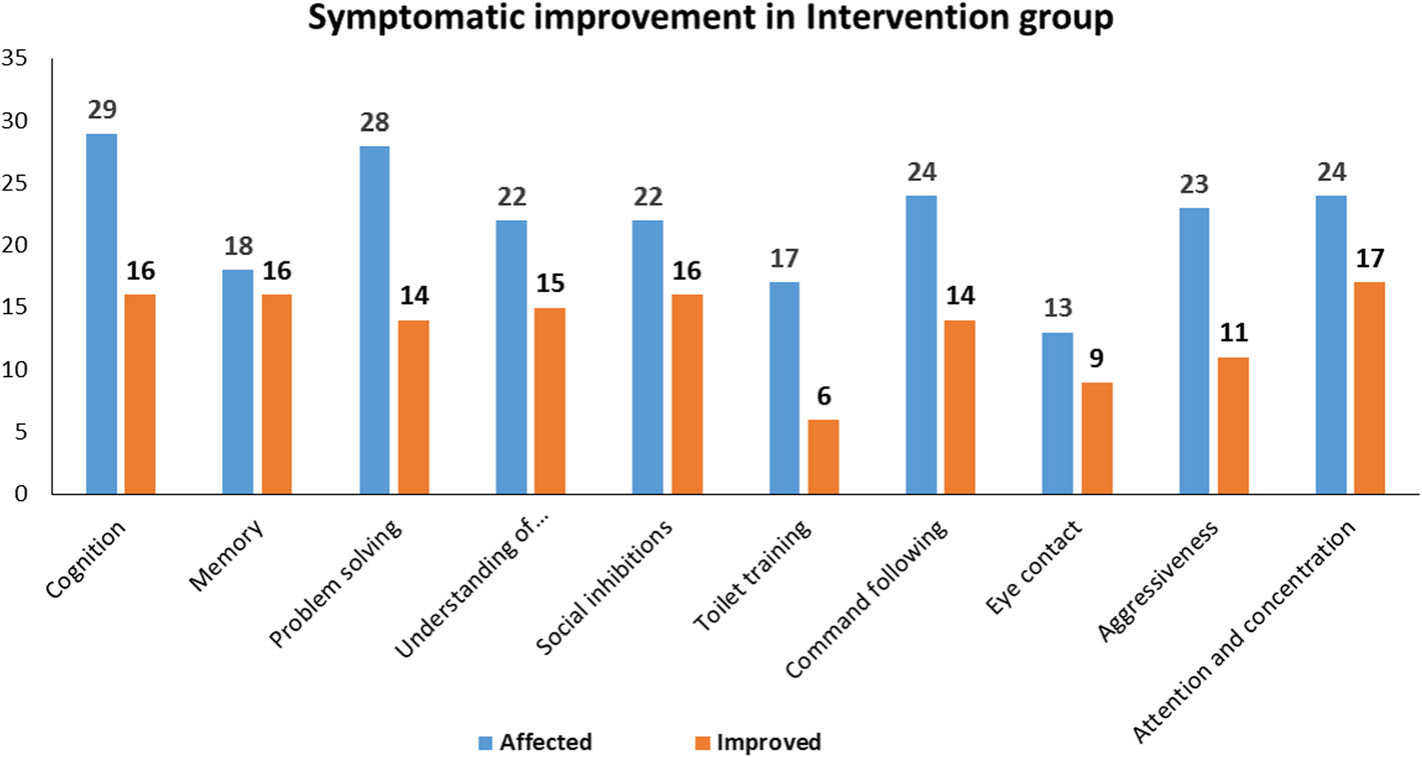
Symptomatic improvements in patients of the intervention group with ID 6 months after cellular therapy

During the symptomatic analysis at 6-month follow up, patients in the intervention group showed improved cognition (54%), memory (64.7%), problem-solving (36%), understanding of relationships (36.36%), social inhibitions (38.63%), toilet training (23.52%), command-following (60.52%), eye contact (57.14%), aggressive behaviour (26.82%) and attention and concentration (50%) (Fig. 1 and Table 2). All of the symptomatic improvements were statistically significant on performing McNemar’s test.

**Table 2:** Statistical analysis for each symptomatic improvement in ID patients in the intervention group using McNemar’s test

Symptom	Number of patients affected	Number of patients improved	Percentage of improvement	McNemar’s test value	*P* value	Significance
Cognition	29	16	55.17	15.015625	0.000107	Significant
Memory	18	16	88.88	15.015625	0.000107	Significant
Problem-solving	28	14	50	13.017857	0.000309	Significant
Understanding of relationships	22	15	68.18	14.016667	0.000181	Significant
Social inhibitions	22	16	72.72	15.000000	0.000108	Significant
Toilet training	17	6	35.29	5.041667	0.024745	Significant
Command-following	24	14	58.33	13.017857	0.000512	Significant
Eye contact	13	9	69.23	8.027778	0.004607	Significant
Aggressiveness	23	11	47.82	10.022727	0.001546	Significant
Attention and concentration	24	17	70.83	16.014706	0.000063	Significant

##### Symptomatic improvements in the rehabilitation group

**Fig. 2:**
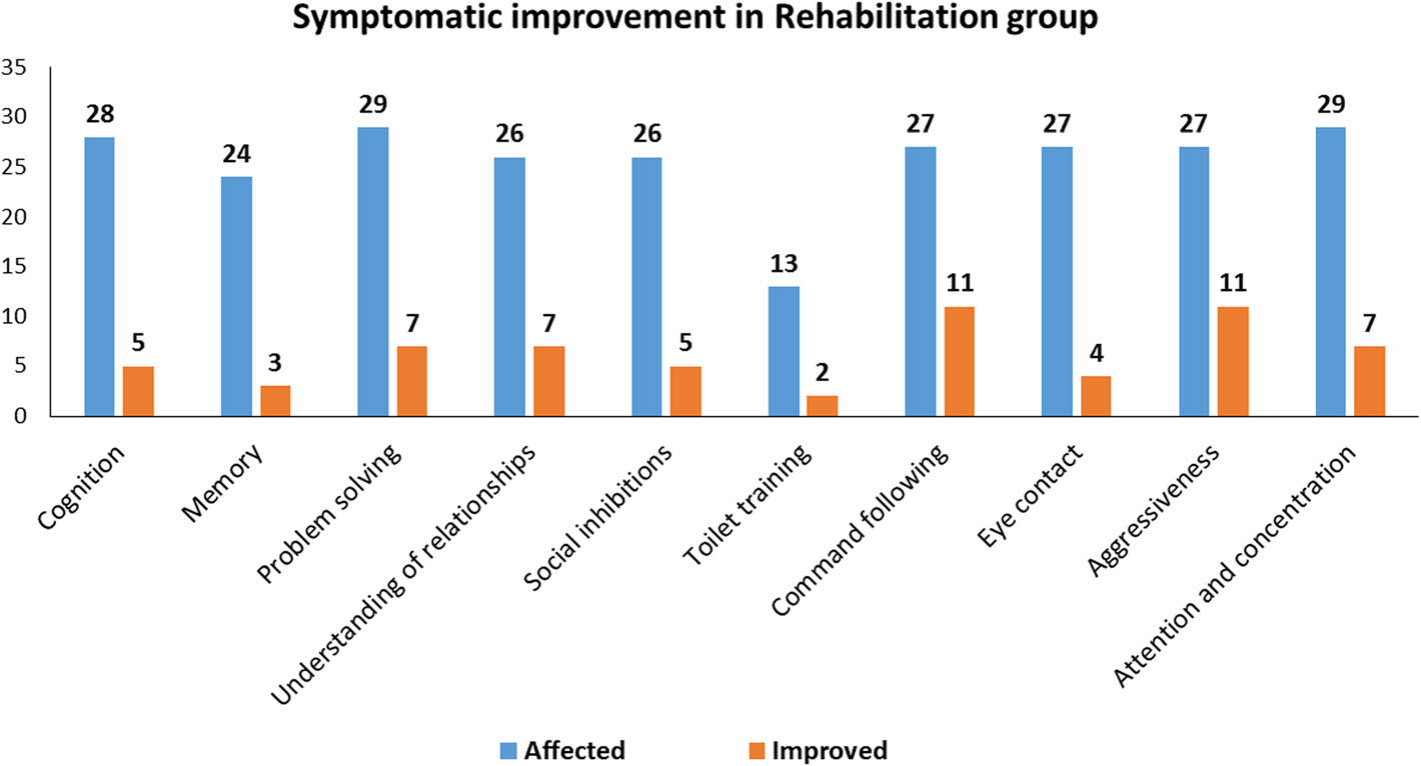
Symptomatic improvements in patients with ID who underwent only a rehabilitation regime (rehabilitation group)

In the rehabilitation group, the percentage improvement in the symptoms was comparatively less than for the intervention group. An improvement of 17.85% in cognition, 12.5% in memory, 24.13% in problem-solving, 26.92% in understanding of relationships, 19.23% in social inhibitions, 15.38% in toilet training, 40.74% in command-following, 14.81% in eye contact, 40.74% in aggressive behaviour and 24.13% in attention and concentration was noted (Fig. 2 and Table 3). However, improvements in cognition, memory, social inhibition, toilette training and eye contact were not statistically significant on performing McNemar’s test.

**Table 3:** Statistical analysis for each symptomatic improvement in ID patients in the rehabilitation group using McNemar’s test

Symptom	Affected	Improved	Percentage of improvement	McNemar’s test value	*P* value	Significance
Cognition	28	5	17.85	3.2	0.0736	Not significant
Memory	24	3	12.5	1.333	0.2482	Not significant
Problem-solving	29	7	24.13	5.143	0.0233	Significant
Understanding of relationships	26	7	26.92	5.143	0.0233	Significant
Social inhibitions	26	5	19.23	3.2	0.0736	Not significant
Toilet training	13	2	15.38	0.5	0.4795	Not significant
Command-following	27	11	40.74	9.091	0.0026	Significant
Eye contact	27	4	14.81	2.25	0.1336	Not significant
Aggressiveness	27	11	40.74	9.091	0.0026	Significant
Attention and concentration	29	7	24.13	5.143	0.0233	Significant

##### Comparison of symptomatic improvements between the intervention and rehabilitation groups

**Fig. 3:**
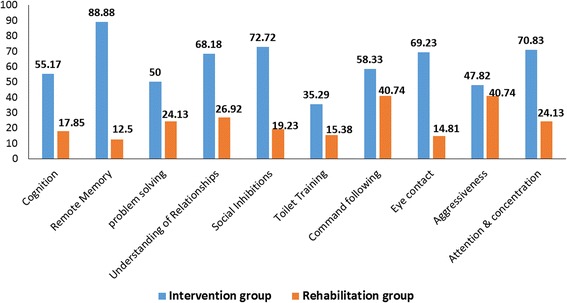
Comparison of overall percentage improvements in the symptoms of ID between the intervention group and the rehabilitation group

To distinguish between the effect of the cellular therapy along with multidisciplinary rehabilitation and that of rehabilitation alone, we performed the percentage analysis for each symptom in both groups. The intervention group demonstrated a better percentage improvement in each of the symptoms (Fig. 3 and Table 4).

**Table 4:** Comparison of symptomatic improvements and statistical analysis between the intervention and rehabilitation groups

Symptom	Intervention group		Rehabilitation group	
	Percentage of improvement	Significance	Percentage of improvement	Significance
Cognition	55.17	Significant	17.85	Not significant
Memory	88.88	Significant	12.5	Not significant
Problem-solving	50	Significant	24.13	Significant
Understanding of relationships	68.18	Significant	26.92	Significant
Social inhibitions	72.72	Significant	19.23	Not significant
Toilet training	35.29	Significant	15.38	Not significant
Command-following	58.33	Significant	40.74	Significant
Eye contact	69.23	Significant	14.81	Not significant
Aggressiveness	47.82	Significant	40.74	Significant
Attention and concentration	70.83	Significant	24.13	Significant

##### Comparison of degree of improvements in the intervention and rehabilitation groups

**Fig. 4:**
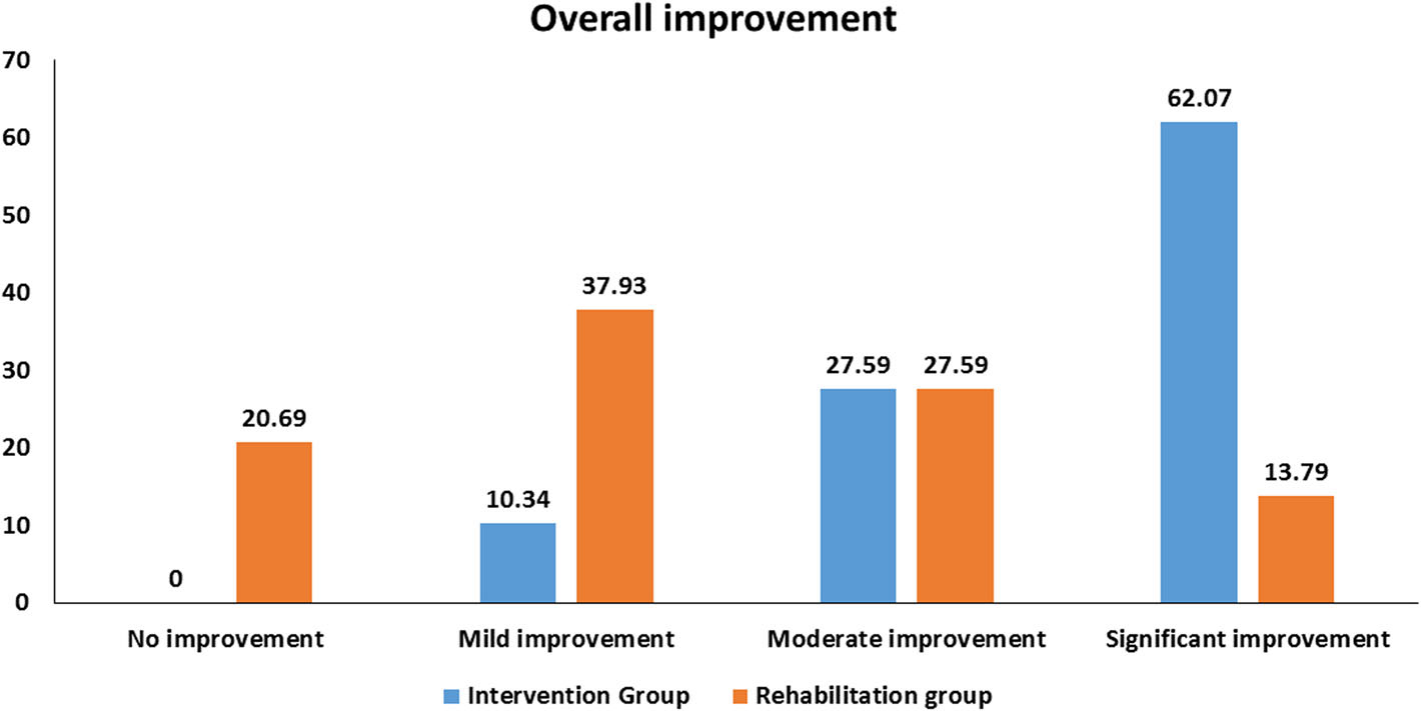
Comparison of overall percentage improvements in ID between the intervention group and the rehabilitation group

On the grading system (as already described), more patients in the intervention group showed significant improvement. In the intervention group, 10.34% of cases showed mild improvement, 27.59% showed moderate improvement and 62.06% showed significant improvement (Fig. 4). In the rehabilitation group, 20.69% of cases showed no improvement, 37.93% showed mild improvement, 27.59% cases showed moderate improvement and 13.79% showed significant improvement (Fig. 4).

#### Intragroup analysis

##### Outcome measures in the intervention group

**Fig. 5:**
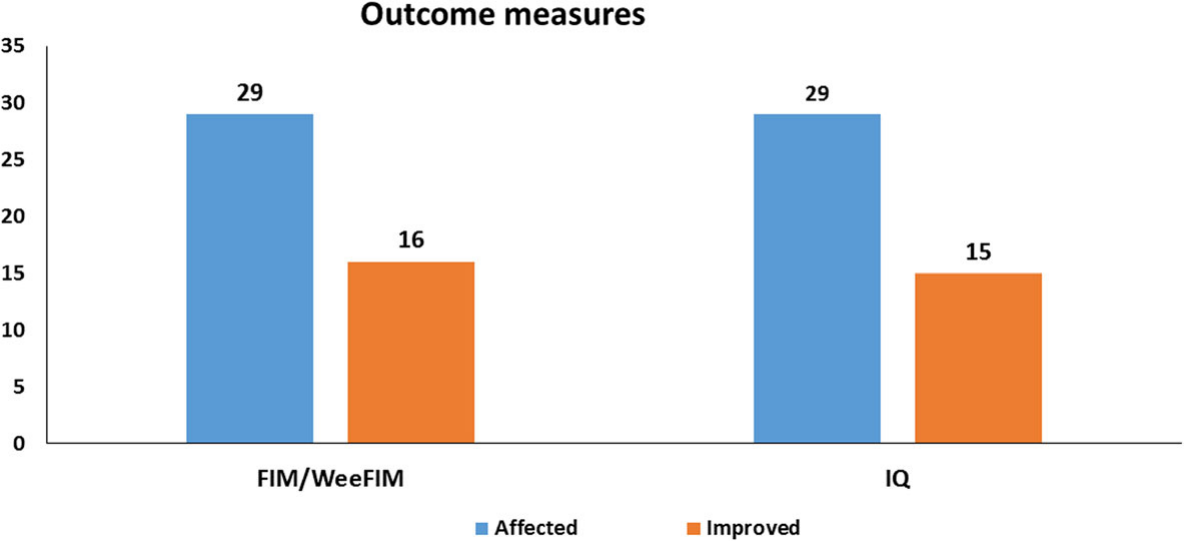
Improvements in outcome measures in patients with ID in the intervention group, 6 months after cellular therapy. FIM Functional Independence Measure, IQ intelligence quotient

The outcome measures showed statistically significant improvement in IQ and FIM/Wee-FIM in the intervention group (Table 5 and Fig. 5).

**Table 5:** Statistical analysis for improvement in outcome measures in ID patients in the intervention group using McNemar’s test

	Affected	Improved	% improvement	McNemar’s test value	*P* value	Significance
FIM/Wee-FIM	29	16	54	16.00093	<0.05	Significant
IQ	29	15	50	15.00926	<0.05	Significant

*FIM* Functional Independence Measure, *IQ* intelligence quotient

Wilcoxon’s matched-pairs signed-rank test showed statistically significant improvement in mean FIM/Wee-FIM scores before and after cellular transplantation (Table 6).

**Table 6:** Comparative analysis of FIM in patients before and after cell therapy using Wilcoxon’s matched-pairs signed-rank test (*N* = 29)

	Mean pre FIM	Mean post FIM	Significance (*P* < 0.05)	*Z* value
FIM score	69.39	75.95	<0.05	–4.0145

*FIM* Functional Independence Measure

##### PET-CT study

**Fig. 6:**
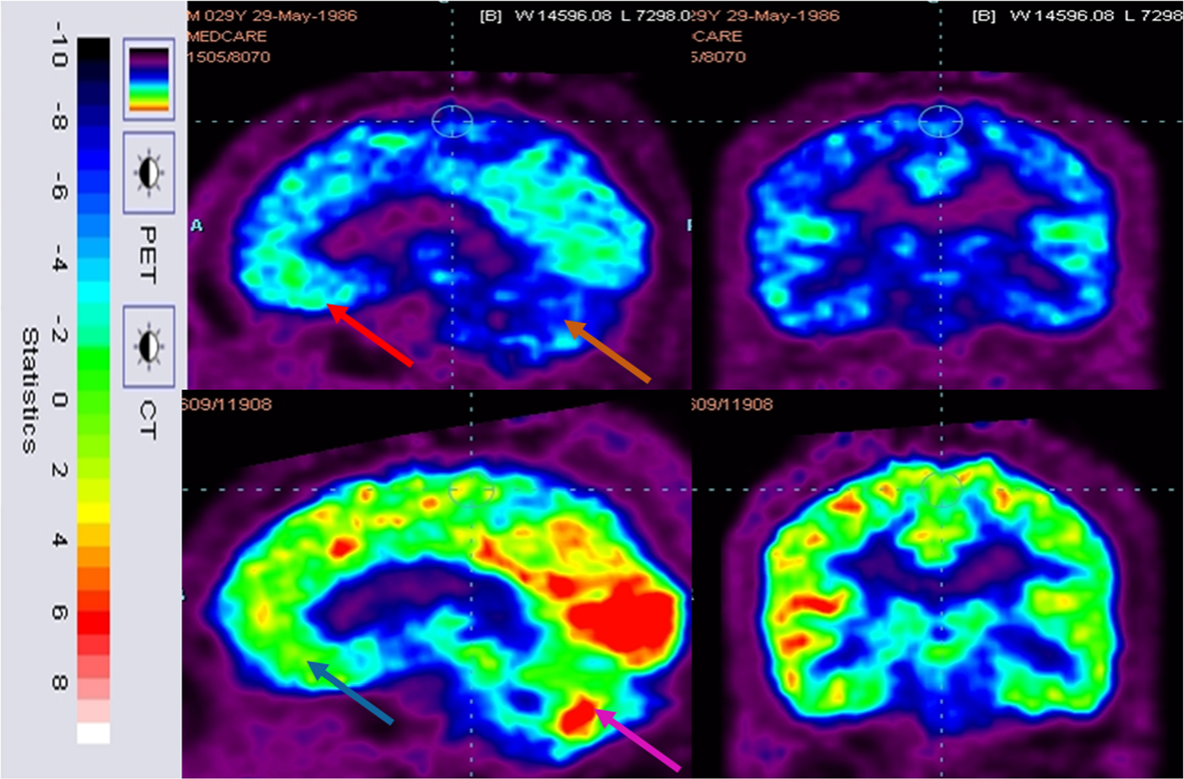
Top row: 18 F-FDG image before cellular therapy showing reduced metabolism in the prefrontal, frontal (red arrow) and cerebellum (brown arrow). Bottom row: improved 18 F-FDG metabolism after cellular therapy metabolism in the prefrontal, frontal (blue arrow) and cerebellum (pink arrow). CT computed tomography, PET positron emission tomography

**Fig. 7:**
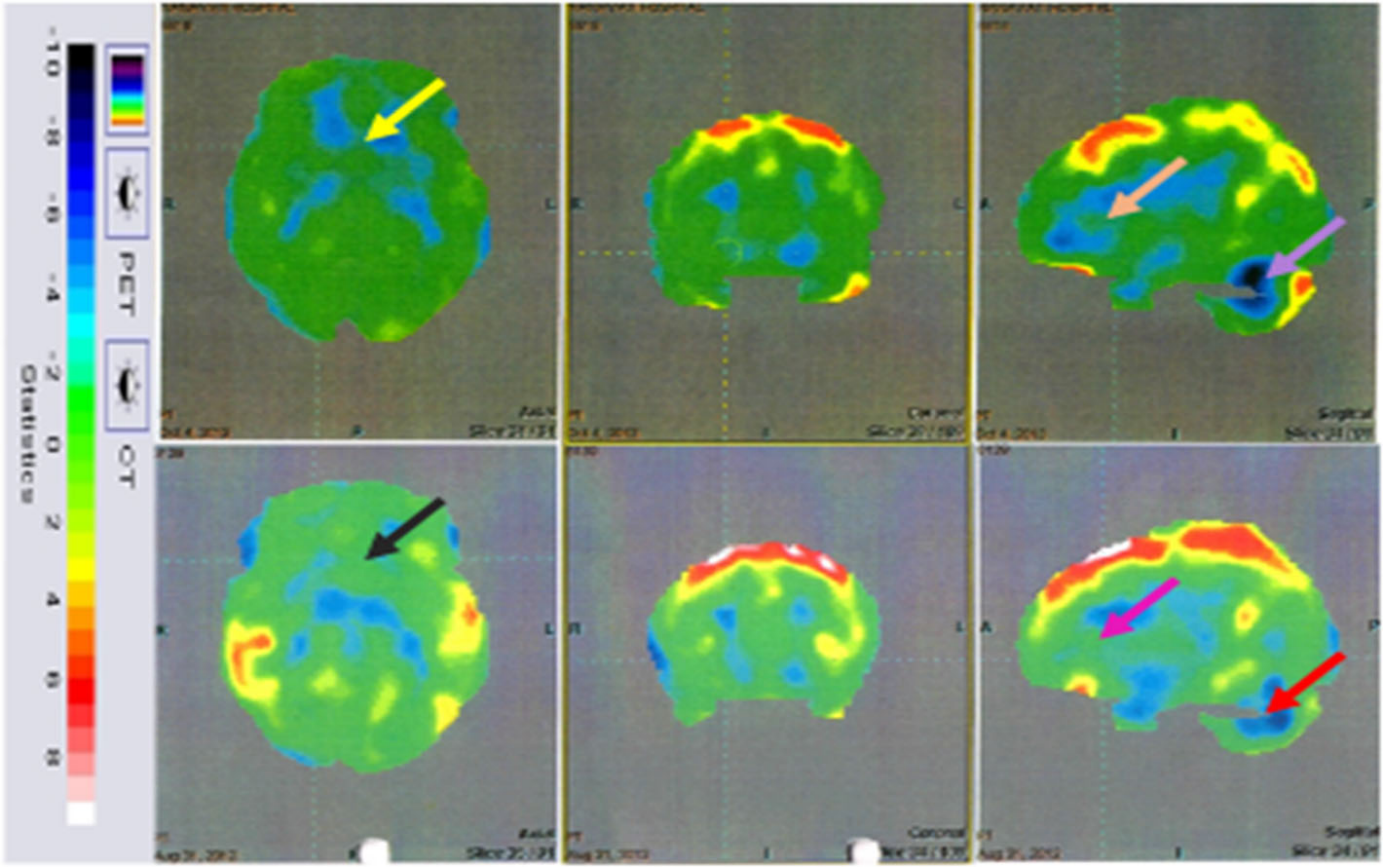
Top row: 18 F-FDG image before cellular therapy showing reduced metabolism in the thalamus (yellow arrow), frontal lobe (orange arrow) and cerebellum (purple) arrow). Bottom row: improved 18 F-FDG metabolism after cellular therapy metabolism in the thalamus (black arrow), frontal lobe (pink arrow) and cerebellum (red arrow). CT computed tomography, PET positron emission tomography

**Fig. 8:**
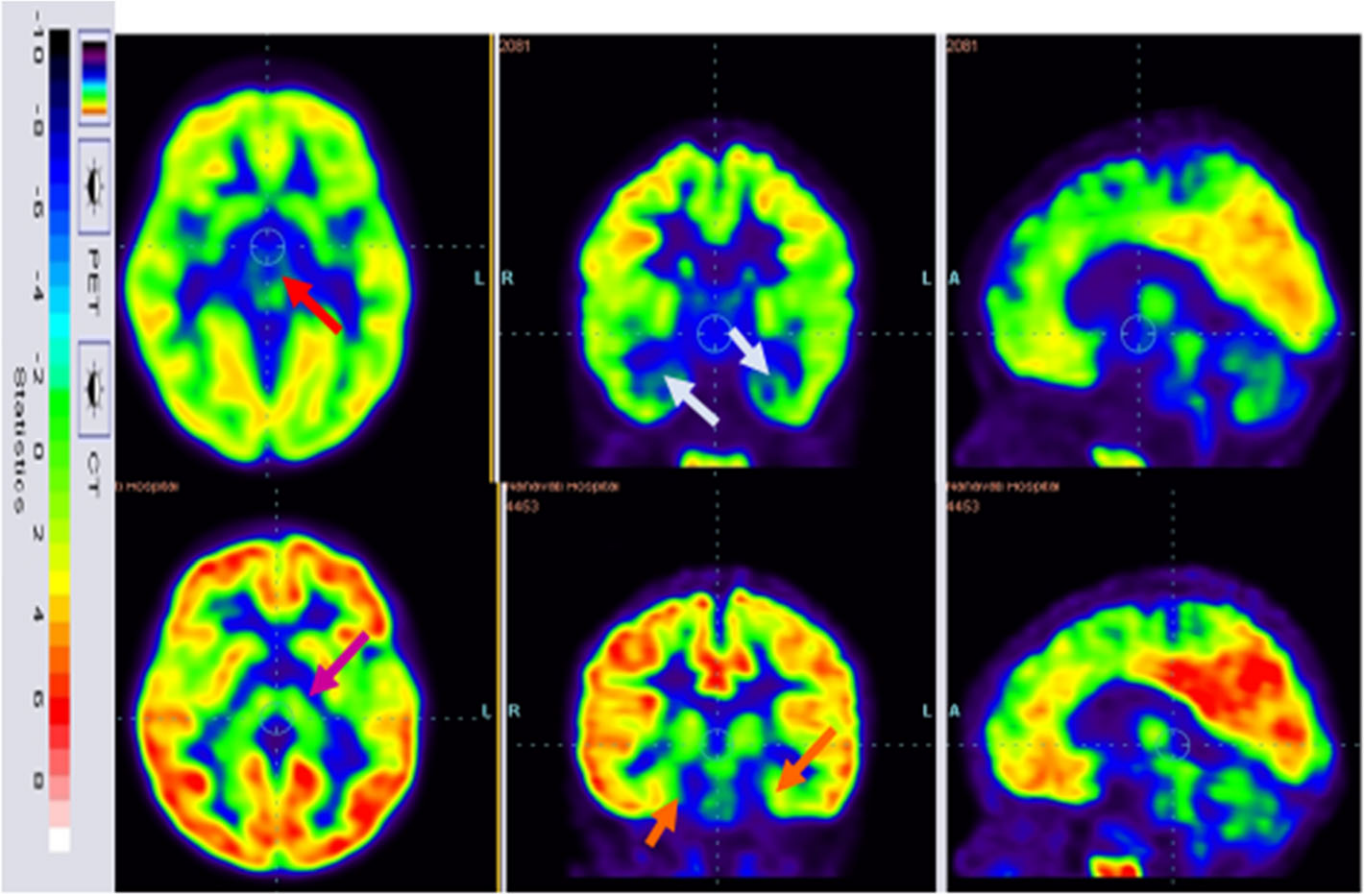
Top row: 18 F-FDG image before cellular therapy showing reduced metabolism in the thalamus (red arrow) and mesial temporal structures (white arrows). Bottom row: improved 18 F-FDG metabolism after cellular therapy metabolism in the thalamus (pink arrow) and mesial temporal structures (orange arrows). CT computed tomography, PET positron emission tomography

PET-CT scans were repeated in three patients of the intervention group at the end of 6 months and they showed improved metabolism after the intervention (Table 7). On comparing the pre-intervention and post-intervention scans, it was observed that the metabolism in areas such as the frontal lobe, parietal cortex, thalamus, mesial temporal structures (amygdala, hippocampus) and cerebellum had increased. The changes were consistent with the clinical and functional improvements demonstrated by these patients (Figs. 6, 7 and 8, summary in Table 7).

**Table 7:** Areas of the brain showing increased metabolism in the PET scan performed in three patients corresponding to functional improvements

Patient	Age (years)/gender	Areas of brain showing improvement in PET	Corresponding improvements observed
1	15/male	Frontal	Planning, problem-solving, command-following, cognitive skills, emotions
		Mesial temporal region	Social participation, learning
		Cerebellum	Balance and coordination
2	15/female	Cerebellum	Balance, coordination and fine motor activities
		Frontal lobe	Command-following, understanding, planning, problem-solving
3	13/female	Frontal lobe	Learning ability, cognitive skills, decision-making
		Amygdala	Social interaction, behaviour
		Thalamus	Sensory interpretation, sleep and consciousness

*PET* positron emission tomography

##### Adverse events

In the intervention group, there were no adverse events recorded at the time of the procedure. During the hospital stay, however, a few patients did show minor procedure-related adverse events: one patient had high-grade fever and three patients had headache and vomiting. These events were self-limiting and relieved within 1 week using medications.

##### Factors affecting the outcome of intervention

It is postulated that the age of the patient and the severity of disorder may affect the clinical outcome of cellular therapy. To analyse the effect of these factors, an analysis was performed on the data for 6 months in the intervention group.

On analysing the age at intervention, it was found that more patients in the paediatric age group showed significant improvement (Table 8). On comparison between the paediatric and adult age groups, the mean percentage improvement in symptoms (58.62% vs 41.37%) was noted to be greater in paediatric patients.

**Table 8:** Number of patients showing improvements based on age of the patients 6 months after cellular therapy

Characteristic	Mild improvement	Moderate Improvement	Significant improvement
Age			
<18 years (paediatric)	1	3	13
≥18 years (adult)	1	3	8

**Fig. 9:**
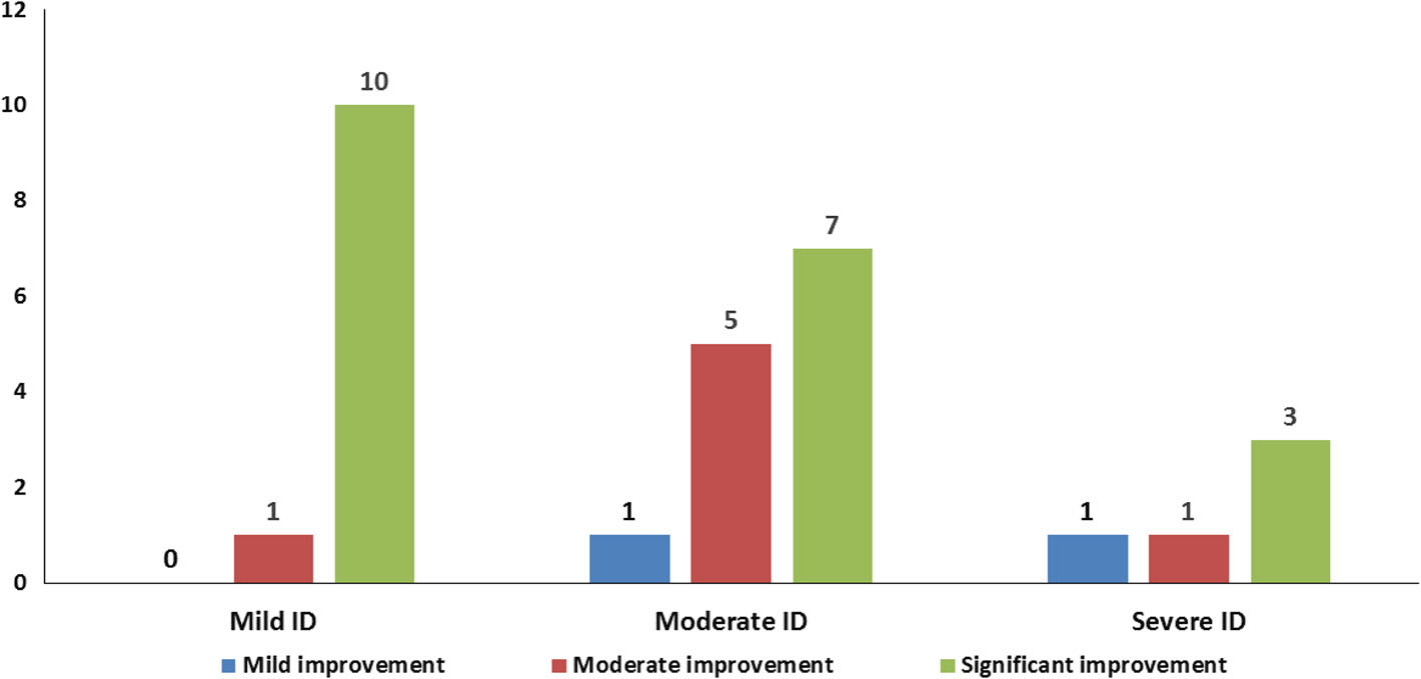
Comparison of improvement in patients in the intervention group with severity of intellectual disability (ID)

On analysing the effect of severity of ID on the clinical outcome of cellular transplantation, more significant improvements were observed in mild cases of ID as compared to moderate and severe ID (Fig. 9, Table 9).

**Table 9:** Improvements in different severity of intellectual disability (ID)

Severity of ID	Mild ID	Moderate ID	Severe ID
Mild improvement	0	1	1
Moderate improvement	1	5	1
Significant improvement	10	7	3

## Discussion

ID is a developmental disorder characterised by cognitive impairment with an onset during early childhood [14]. The aetiology of ID is heterogeneous, including premature birth, gene mutation and chromosomal abnormalities (Trisomy 21 and fragile X), toxins, prenatal infections and environmental factors (malnutrition, emotional and social deprivation) [14, 15].

### Pathophysiology of ID

ID is a highly diverse disorder in terms of the severity of the cognitive disability and the manifestation of other non-cognitive symptoms, which can be related partly to the heterogeneity in the underlying causes [16]. Neural dysfunction underlying ID may include reduction in neuron numbers, disturbed neuronal migration and alterations in dendritic arborisation and morphology [17]. Neuropathological studies of post-mortem brains of persons with ID have shown that the symptoms are usually associated with detectable alterations in the structure of the cerebral cortex, hippocampus and/or various other brain areas [18]. During postnatal brain development, experience-dependent synaptic rearrangement is crucial to optimise neuronal network circuitry to meet environmental demands [19]. ID could ensue from interference with this process and result in a limited ability of the brain to process information.

### Classification of ID

According to DSM V, the four revised severity specifiers have been stated based on the level of adaptive functioning and not only IQ. Individuals with an IQ of 55–70 belong to mild ID; those with IQ of 40–55 are regarded as having moderate ID; IQ of 25–40 is regarded as severe mental retardation; and those with an IQ lower than 25 are considered to have profound ID. There is a classification of “unspecified intellectual disability” which describes individuals’ functioning when the degree of severity cannot be judged due to various reasons such as locomotor disability, severe behavioural problems, sensory impairments and so forth [20].

Severe forms of MR are often associated with brain malformations, microcephaly and/or neuronal migration deficits which limit the capacity to process information [21]. Milder forms of MR show abnormal changes in brain anatomy, including relevant areas like the cerebral cortex and hippocampus [22].

### Rationale for cellular therapy

Conventional treatments such as behavioural and cognitive therapies focus on treating the behavioural issues, aggression or self-injurious behaviours that are associated with ID [23]. But these modalities do not address the underlying neural dysfunction. The population of patients with ID are intellectually and functionally dependent on caretakers and are considered a socioeconomic burden in society. Hence, there is a critical need to find new avenues for management of ID which focuses on the underlying cause of the cognitive deficit, making the affected population functionally independent. Cellular therapy has shown promise to treat the neuronal damage through neurorestorative and neuroprotective mechanisms in many clinical studies [24, 25]. To study the therapeutic potential and safety of cellular therapy in ID, we administered autologous BMMNCs to the patients intrathecally.

Bone marrow-derived cells are advantageous for therapy due to their properties like multipotency self-renewal and transdifferentiation, and can be implanted into the developing and mature CNS [26, 27]. Bone marrow is a rich source of heterogeneous populations of stem cells, including haematopoietic stem cells (HSCs), mesenchymal stem cells (MSCs) and endothelial progenitor cells (EPCs) [28]. This offers great advantage with a variety of effects from different cell types.

### Counteracting mechanism of action of bone marrow mononuclear cells

Cellular therapy harnesses the neurogenic capacity of BMMNCs in order to repopulate and repair the injured brain cells [29]. BMMNCs promote neuroregeneration by multiplying and differentiating into various cells including neural cells and oligodendrocytes. The oligodendrocytes help in remyelination of the damaged axons in the injured brain and repair the neural connections [30].

The MNCs exert reparative effects by homing to the abnormal regions of the brain and expressing paracrine effects through secretion of factors including cytokines and growth factors such as connective tissue growth factor, fibroblast growth factors 2 and 7, interleukins, vascular endothelial growth factor (VEGF), fibroblast growth factor (FGF) and basic fibroblast growth factor (bFGF) which are responsible for cell proliferation [31, 32].

These factors also act like catalysts for the stem cell-driven process by increasing angiogenesis, decreasing inflammation, preventing apoptosis, remodelling the extracellular matrix and activating satellite cells [33]. These cells also stimulate local repair by homing at the site of damage and enhancing proliferation, cell recruitment and maturation of endogenous stem or progenitor cells [31].

### Route of administration

Efficient delivery of cells at the site of injury plays a crucial role during cellular response. Intravenous administration is less invasive but the cells might get entrapped in the pulmonary circulation [34]. Basic animal and clinical experiments advocate use of the intrathecal route or lumbar puncture for cell delivery [35, 36]. The intrathecal route of transplantation is a safe and minimally invasive approach to provide cells to the brain without causing any neural tissue damage. Transplanting cells into the subarachnoid space of the spinal cord mobilises the cells through cerebrospinal fluid (CSF) and allows efficient delivery of cells in the brain [37, 38].

### Importance of rehabilitation

It was observed that the patients who underwent regular rehabilitation regime following cellular therapy showed significant improvement. Many preclinical and clinical studies have proved that voluntary physical exercise induces precursor cell proliferation, thereby expanding the pool and enhancing the mobilisation of progenitor cells that are available for neuroregeneration [39, 40]. It was also observed that rehabilitation along with cellular therapy showed better results than in those patients who underwent only rehabilitation. This may suggest that exercise further enhances the effects of cellular therapy.

### Clinical outcome of this study

The clinical outcome seen in the intervention group is evidence for the concept of application of cellular therapy in ID. In the present study, all patients had undergone the standard methods of treatment available and still demonstrated the residual deficits before undergoing cellular therapy. Here, the patients in the intervention group showed statistically significant improvements in the areas of cognition, memory, problem-solving, understanding of relations, social inhibitions, toilet training, command-following, eye contact, aggressive behaviour attention and concentration after cellular therapy. These improvements led to the functional improvements and improvements in activities of daily living which were reflected as improved scores of FIM/Wee-FIM. We found that the rehabilitation group showed a lesser percentage improvement in the symptoms as compared to the intervention group.

The improvements in the intervention group can be attributed to the physiological processes occurring at the microcellular level in the brain as a result of cellular therapy. The neurorestorative effects exerted by the BMMNCs like angiogenesis, neovascularisation, production of growth factors and paracrine effects lead to improved synaptic connectivity and thereby improved information processing in the damaged brain areas. These processes help in the formation of neuronal circuits, which are strengthened with neurorehabilitation. Therefore, cellular therapy has the potential to repair damaged neural circuits at the molecular, structural and functional levels.

### Comparison between the rehabilitation and intervention groups

Restorative therapies are maximally effective at improving outcomes when introduced in parallel with behavioural reinforcement such as rehabilitation therapy [41]. This was supported by our study results. All patients in the intervention group showed improvements in symptoms associated with ID, whereas 20.69% of patients in the rehabilitation group showed no improvements. Therefore, we conclude that cellular therapy along with rehabilitation played a vital role in the symptomatic improvements seen after the intervention.

### Outcome measures: IQ and FIM

There has been considerable debate regarding the evaluation of intellectual functioning. While IQ is not the only means of evaluating mental capacity for reasoning, learning and problem-solving, it is the most frequent tool used to characterise participants and to assess cognitive ability according to DSM V [20]. IQ gives a relatively reliable picture of the magnitude of the mental deficit in an affected individual and improvement after the intervention [42]. On evaluation, the IQ component showed significant improvement after 6 months of cellular therapy in the intervention group.

FIM/Wee-FIM is used widely and accepted as a functional-level assessment tool that evaluates the functional status of patients throughout the rehabilitation process [43]. The 18 items on the FIM assess the patient’s degree of disability and burden of care. Thirteen items define disability in motor functions and five define disability in cognitive functions [43, 44]. The improvement in the FIM score was significant after the cellular therapy, suggesting that there was a functional improvement post intervention in both the motor and cognitive components.

Overall, these outcome measures suggest that cellular transplantation promotes functional and symptomatic recovery leading to an improved quality of life in ID patients, making them functionally independent.

### PET-CT scan findings

In this study, PET-CT brain scan was used as a monitoring tool to determine changes in the brain metabolism after the intervention. The PET-CT scan provides measures of brain glucose metabolism using tracer [18 F]-fluorodeoxyglucose (FDG) that indirectly correlates with the function of the neurons. Hypometabolism indicates hypofunctionality and hence improvement in function will be seen as increased metabolism (FDG uptake) [45].

Interpretation of the PET-CT scan changes correlated with the clinical improvement in the patients. The improvements observed in social participation and in following commands in the patients can be attributed to improved frontal lobe functioning as identified on the PET scan [46]. Increased FDG uptake in mesial temporal structures correlates with enhanced memory, learning ability, cognitive skills, emotional learning and decision-making [47]. The improvements in balance, coordination and fine motor activities can be attributed to the increased function of the cerebellum as reflected in the PET scan of the patients [48].

### Factors affecting the clinical outcome of cellular transplantation

The effect of age at intervention and severity of ID was analysed to assess their influence on the clinical outcome after cellular transplantation.

#### Importance of age at intervention

In this study it was observed that patients who were ministered at an early age (i.e. < 18 years) showed better improvement than those who were treated at a later stage (≥18 years). One postulated hypothesis is that the neural circuits, which form the base for learning, behaviour and health, are more plastic during the initial years of life and over time they become increasingly difficult to alter [49]. The immature brain may be more amenable than the mature brain to their functional incorporation [50]. There is also an age-related decline in the potency of these cells, which might affect their usefulness in remodelling of the CNS [51].

#### Severity of disorder

It has been observed that the mild cases of ID have a better symptomatic improvement than the moderate and severe cases. In mild cases, recovery can be rapid as axonal function remains intact. In severe cases, axonal degeneration and a greater degree of residual injury is often observed which forbids early recovery [52]. Also, more number of doses may be required to gain functional improvement in severe ID.

## Limitations

The absence of IQ scores in the rehabilitation group was one of the limitations. However, the greater improvements in the symptoms of ID noted in the intervention group suggest that cellular therapy played a vital role in recovery. PET-CT scan used as evidence in a small number of patients was another limitation.

## Conclusion

This proof-of-concept study demonstrates that cellular therapy along with multidisciplinary neurorehabilitation has a better outcome than standard rehabilitation alone. The neurorestorative and neuroregenerative properties of cellular therapy had a vital role in accelerating functional recovery in ID patients. The multiple counteracting mechanisms of BMMNCs promote a reparative process in the dysfunctional brain which was reflected by clinical and functional improvement. This study also reaffirms the safety and efficacy of intrathecal autologous BMMNC therapy in ID. Cellular therapy at a younger age is beneficial, which can be attributed to maximal neural plasticity of the immature brain. Mild cases have a better recovery curve which may be due to intact axonal function. PET-CT scan may be used to observe the metabolic improvements after cellular therapy. To exploit the potential of cellular therapy in ID patients, further large-scale, blinded, randomised clinical trials will be needed. Future studies should consider the use of PET-CT scan as a tool to substantiate the effects of cellular therapy in ID.

## Abbreviations

bFGF: Basic fibroblast growth factor
BMMNC: Bone marrow mononuclear cell
CD34: Cluster of differentiation 34
CSF: Cerebrospinal fluid
DSM V: *The Diagnostic and Statistical Manual of Mental Disorders*, fifth edition
EEG: Electroencephalogram
EPC: Endothelial progenitor cell
FACS: Fluorescence activated cell sorting
FGF: Fibroblast growth factor
FIM: Functional Independence Measure
G-CSF: Granulocyte colony stimulating factor
HBV: Hepatitis B virus
HCV: Hepatitis C virus
HIV: Human immunodeficiency virus
HSC: Haematopoietic stem cell
IC-SCRT: Institutional Committee for Stem Cell Research and Therapy
ID: Intellectual disability
IQ: Intelligence quotient
MRI: Magnetic resonance imaging
MSC: Mesenchymal stem cell
OPD: Outpatient department
PET-CT: Positron emission tomography-computed tomography
VEGF: Vascular endothelial growth factor
